# Estimated glomerular filtration rate based on hospital discharge creatinine may significantly overestimate renal function and underestimate chronic kidney disease in survivors of critical illness

**DOI:** 10.1186/cc12356

**Published:** 2013-03-19

**Authors:** I Kolic, J Purdell-Lewis, CJ Kirwan, JR Prowle

**Affiliations:** 1 Royal London Hospital, Barts Health NHS Trust, London, UK

## Introduction

Acute kidney injury (AKI) complicates over 50% of ICU admissions. Episodes of AKI are a major risk factor for development or progression of chronic kidney disease (CKD); however, methods of estimated glomerular filtration rate (eGFR) may be poorly calibrated to survivors of critical illness who may have reduced muscle mass. We hypothesized that eGFR may underestimate rates and severity of CKD in ICU survivors.

## Methods

A retrospective observational study of renal function in all patients admitted to a London teaching hospital ICU for ≥5 days and surviving to hospital discharge in 2011. We excluded cases with current or new diagnosis of end-stage renal disease or renal transplant. We assessed AKI in ICU by KDIGO 1 criteria and hospital discharge eGFR by the CKD-EPI equation. For comparison we assumed a normal GFR in a healthy individual as 120 ml/minute/1.73 m^2 ^at age 20 decreasing by 0.8 per year over age 20.

## Results

**Figure 1:**
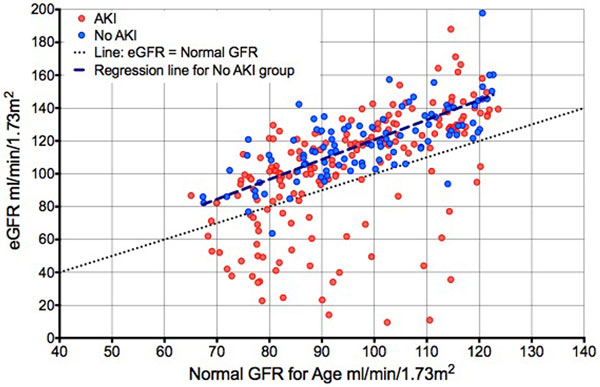
**eGFR at hospital discharge versus expected normal GFR for age in 280 ICU survivors**.

We identified 282 patients, 180 of whom had AKI. Median age was 50 and 68% were male. Median hospital discharge serum creatinine was 573 μmol/l (range 16 to 654), median eGFR was significantly higher than predicted normal GFR for age at 115 versus predicted 95 (*P *0.001, median difference 16). In patients who had not had AKI discharge the eGFR was 119 versus normal predicted 98 (*P *0.001, median difference 19), suggesting that eGFR could be overestimating true GFR in our population by at least a factor of 1.23 (Figure 1). Applying this correction factor to eGFRs of patients who had recovered from AKI resulted in 44% more diagnoses of CKD (eGFR <60) at hospital discharge (36 vs. 25).

## Conclusion

eGFR may overestimate renal function in survivors of critical illness confounding identification of CKD in this at-risk population. Prospective studies with measurement of actual GFR are required to assess the burden of CKD in survivors of critical illness.

